# A Novel Method for Effective Cell Segmentation and Tracking in Phase Contrast Microscopic Images

**DOI:** 10.3390/s21103516

**Published:** 2021-05-18

**Authors:** Hongju Jo, Junghun Han, Yoon Suk Kim, Yongheum Lee, Sejung Yang

**Affiliations:** 1 Department of Biomedical Engineering, College of Software and Digital Healthcare Convergence, Yonsei University, Wonju 26493, Korea; jhjoo3217@yonsei.ac.kr (H.J.); cheque@yonsei.ac.kr (J.H.); koaim@yonsei.ac.kr (Y.L.); 2 Department of Biomedical Laboratory Science, College of Software and Digital Healthcare Convergence, Yonsei University, Wonju 26493, Korea; yoonsukkim@yonsei.ac.kr

**Keywords:** cell migration, phase contrast microscope, segmentation, tracking, noise reduction

## Abstract

Cell migration plays an important role in the identification of various diseases and physiological phenomena in living organisms, such as cancer metastasis, nerve development, immune function, wound healing, and embryo formulation and development. The study of cell migration with a real-time microscope generally takes several hours and involves analysis of the movement characteristics by tracking the positions of cells at each time interval in the images of the observed cells. Morphological analysis considers the shapes of the cells, and a phase contrast microscope is used to observe the shape clearly. Therefore, we developed a segmentation and tracking method to perform a kinetic analysis by considering the morphological transformation of cells. The main features of the algorithm are noise reduction using a block-matching 3D filtering method, k-means clustering to mitigate the halo signal that interferes with cell segmentation, and the detection of cell boundaries via active contours, which is an excellent way to detect boundaries. The reliability of the algorithm developed in this study was verified using a comparison with the manual tracking results. In addition, the segmentation results were compared to our method with unsupervised state-of-the-art methods to verify the proposed segmentation process. As a result of the study, the proposed method had a lower error of less than 40% compared to the conventional active contour method.

## 1. Introduction

Cellular dynamics are important with respect to many biological processes that directly affect human health [1]. Cell migration is a basic cell function that is the source of cell life and plays a fundamental role in various diseases [2] and physiological phenomena, such as cancer metastasis, nerve development, immune function, wound healing, and embryo formulation [3]. Thus, analysis of the migration characteristics and motility of cells is essential for physiological and pathological research [4,5,6]. Analysis of the migration characteristics of metastatic cancer cells is essential for detecting the deterioration of cancer cells, and as a basic study, an analysis of the individual motility of each cell is being conducted [7]. In the case of immune cells, studies on the effectiveness of antigen targeting and the individual motility of cells to enhance the effectiveness are being conducted [8]. These eukaryotic cells have different motor forms and properties, and although they are affected by different types of extracellular matrixes, the types of feet that the cells use to exercise, such as the pseudopodium, characterize their basic motility [9]. The study of these cell migration characteristics is generally conducted through probabilistic model analyses, such as persistent random walk and levy walk, by tracking the positions of cells at each interval in the images of the observed cells after several hours of taking a picture with a real-time microscope [7,10]. Although cell tracking is an effective method for quantitative analysis of cell migration characteristics, this requires a lot of effort and cost. Therefore, many studies have been conducted on automatic cell tracking methods [11,12,13].

Recently, research has been conducted on the analysis of migration characteristics based on the morphological transformation of cells [14]. When cells migrate, they move through the pseudopodium and lamellipodia, where the shapes of the cells change in various ways, and studies are being conducted to predict mobility through the probabilistic calculation of the direction or shape in which they extend [7,15]. Thus, an important goal is to change the dynamic properties of cells, for example, by suppressing the motility of parasites. In addition, morphological changes in cells play an important role in the phagocytosis of immune cells in the antigen or host cells by parasites in the “immune synapses” [16]. To proceed with these studies, it is necessary to observe the shape of a cell precisely. Additionally, these depend on accurate information about the cell positions, requiring computational shape segmentation and tracking methods. Moreover, manual segmentation or tracking is avoided because these studies analyze a large number of cell images and require a high level of precision. Since manual segmentation is subjective, errors can occur, especially when analyzing cell movement characteristics at short intervals, resulting in relatively large errors.

To perform a kinetic analysis considering the morphological transformation of cells, it is necessary to detect and consider all the pseudopodium, podosomes, etc., to determine the shape of the entire cell. A microscope is needed to observe these structures, and phase contrast microscopes can be used to visualize a particular structure (such as a filament) [17]. Thin, transparent areas, such as the pseudopodium, can be observed more clearly [18]. Therefore, phase-contrast microscopic images are utilized in many cell migration studies because they are easy to analyze using images [19].

However, in phase contrast microscopy, a favorable high contrast at the cell boundary leads to problems, such as halo patterns, which can complicate cell segmentation [20]. While segmentation techniques are becoming increasingly common in the field of fluorescence microscopy, less accurate and robust methods have been developed for segmenting in phase-contrast images. Several methods have been developed for detecting and counting or tracking cells in a phase contrast image, but the focus has not been on detecting the shapes of cells [21,22,23,24,25,26,27,28,29]. In particular, there are not many studies that have conducted precise segmentation considering halo effects and it is rare to fundamentally eliminate the halo effect itself [30,31,32,33]. State-of-the-art techniques include deep learning-based supervised learning methods that are learned with correct answers and unsupervised methods using threshold values. Since our task is an unsupervised learning method, we compare the Empirical Gradient Threshold [29] (EGT) method using image histogram and the phase contrast microscopy segmentation toolbox [33] (PHANTAST) method using local contrast thresholding and halo effect removal with the proposed method.

It is necessary to obtain a boundary to detect the shape of an object in an image. Several segmentation methods, including active contour, which is a precise high-performance technique, have been recently employed. In addition to the cell detection, the active contour method is widely used as a method for detecting objects in various fields such as skin disease detection, tumor detection, heart detection, etc. [34,35]. In our study, the active contour model was adopted to precisely segment and detect the boundary of cells. However, since the active contour method requires an initial point for each object, it is difficult to implement when the number of cells is large. Therefore, we are limited to single cell research.

When an active contour method is applied to phase contrast microscopic images, it is difficult to distinguish between the cell boundaries and the halo. The contrast varies depending on the thickness or substance of the boundary in a cell, which makes the halo effect less constant [36]. Because of this, non-uniform results are obtained while calculating the energy value of the boundary required by the active contour. Therefore, this study proposes a novel method to eliminate the halo effect itself to solve this halo effect. Using the K-means clustering method, which is a machine learning (ML) method that is mathematically simple and rapidly calculated and converged and can be easily implemented [37], only the halo effect is extracted and removed. By eliminating halo effects, the active contour model, which guarantees excellent performance for precise segmentation, but was difficult to use due to its dependence on initial value, was easily available and precise segmentation was performed. The K-means clustering method is particularly widely used for classification purposes in data statistics and analysis studies, and also for image segmentation purposes such as cell nucleus extraction and white blood cells extraction in imaging fields [38]. It is mainly used for the purpose of obtaining a desired target, but our study shows a new applicability in that it was used as a pre-processing method to remove specific problem phenomena.

In summary, the contributions of our proposed work are as follows:

1. An active contour model showing strong performance in shape detection was used for acquiring the shape of the cell in a phase contrast microscope.
2. Since the halo effect that occurs in the phase contrast microscope interferes with the precise segmentation of the cell shape, we propose a solution to remove it.
3. The conventional methods have performed the segmentation for the cell boundary through various complex image processing techniques that distinguish between the halo effect and the cell boundary. In this work, we propose a novel method that uses the ML technique K-means clustering method to separate and correct halo effects from the background signals and cell boundaries, eliminating the basic problem cause itself after denoising.
4. The method of this study, which performed segmentation by removing the halo effect, was verified by comparing two methods, the method performed by the manual method, which is a basic method and is used a ground truth for the proposed method, and the method performed by segmentation without removing the halo effect.
5. The results ensure the novelty and reliability of the method proposed in this study.

In the following chapters, we described the process through which the shapes of cells can be accurately detected. First, the noise in the microscope image is removed, and background correction is utilized to mitigate the halo signal. Second, the shape of the cell is detected using the active contour method, followed by the center of mass. Based on the manual tracking results, we compared the tracking results of the images where the halo signal was not removed with those where it was removed and verify the reliability of the algorithms developed in this study. Furthermore, we verified the effectiveness of segmentation through comparisons with other state-of-the-art techniques. Finally, the paper concludes with future works.

## 2. Materials and Methods

### 2.1. Sample Preparation and Experiment

The immortalized line of human T lymphocyte cells, Jurkat cells, was selected for tracking. The Jurkat cells were cultured in Roswell Park Memorial Institute 1640 medium (Gibco, Grand Island, New York, USA), supplemented with 10% (*v*/*v*) fetal bovine serum (Gibco, Grand Island, New York, USA), 1% (*v*/*v*) penicillin–streptomycin (Invitrogen, Carlsbad, California, USA), 1% 1M HEPES (Gibco, Grand Island, New York, USA), 1% MEM non-essential amino acid solution (Gibco, Grand Island, New York, USA), and 1% sodium pyruvate (Gibco, Grand Island, New York, USA). They were subsequently incubated at 37 °C in 5% CO_2_.

After attaching the Jurkat cells to a 60∅ dish coated with fibronectin human plasma (Sigma, St. Louis, Missouri, USA) at 20 μg/mL, the camera started capturing it after 30 min of stabilization. A confluency of 2% was used to ensure a sufficient distance between cells. Before stabilizing the cell, a live cell microscopic device (OKOLAB, Ottaviano, NA, Italy) was employed for sufficient heating of the device. A Nikon TE1000 microscope with a 10× phase contrast microscope (Nikon, Minato-ku, Tokyo, Japan) was utilized for image cell migration using a charge-coupled device camera. Images were taken every 2 min for 4 h, and we used the perfect focus system in the microscope to avoid the defocus problem from long-lasting capture.

### 2.2. Image Acquisition and Processing

**Figure 1:**
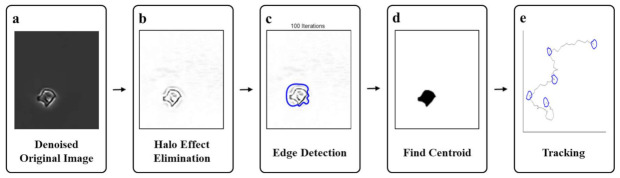
Entire pipeline of the cell segmentation and tracking algorithm. (**a**) is a denoised original image, which is obtained using the method described in the next section. (**b**) shows Halo effect elimination image. After denoising, the Halo effect remains, and Halo effect elimination is performed. (**c**) is the edge detection process. (**d**) shows the center point finding for cell tracking. (**e**) shows the cell tracking result using the proposed methods.

Image processing algorithms were implemented using the MATLAB and Image Processing Toolbox Release 2019b. Multiple cell images, taken using phase-contrast microscopes, were saved by cropping each individual cell. A tracking process was conducted for each cell. The entire pipeline, i.e., the process of cropping an image from the entire image to each cell described earlier, is presented in Figure 1.

#### 2.2.1. Denoising

In microscopic images, noise can be modeled mainly as a Poisson and Gaussian distribution. Gaussian noise can be modeled as an additive and an independent form in the images, whereas Poisson noise can be modeled as a multiplicative and image-dependent form, which is difficult to handle. To eliminate these noises, three steps were performed.

**Figure 2:**
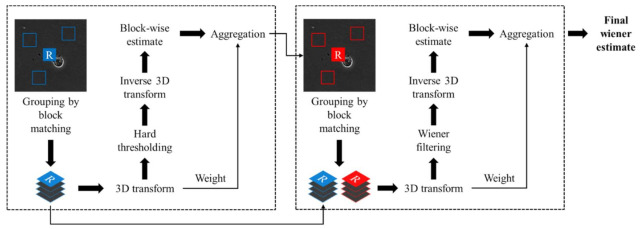
BM3D flowchart. Adapted from ref. [41].

The first step is noise estimation, which estimates the variance of Gaussian noise and Poisson noise [39]. In the noise estimation step, the image is divided into several patches, and each patch’s noise component and variance are calculated locally. By fitting this result to the estimated value of the noise component of the original image, the total noise component can be estimated. The following step is an Anscombe transformation [40] step, which is a variance-stabilizing transformation. Through the Anscombe transformation, the variance of the Poisson noise is fixed at a constant value, such as the variance of Gaussian noise. Therefore, scanning transmission electron microscopy images can be modeled to have Gaussian noise only. The final step is block matching 3D (BM3D) filtering, an effective denoising method for Gaussian noise [41]. Figure 2 presents a flowchart of BM3D filtering. BM3D filtering consists of two main stages.

The first stage is to organize images into blocks, group them into a 3D formation, and co-filter them to create images for use in the second stage. The distance between the blocks is calculated, and blocks smaller than a certain thresholding value are set to similar blocks. The distance between the blocks can be calculated as follows:

(1) $$Dist^{noisy}\;\left(B_{x_{R}},B_{x}\right)=\frac{{\left\|B_{x_{R}}-B_{x}\right\|}_{2}^{2}}{{\left(N_{1}^{ht}\right)}^{2}}$$

where ${\left\|\cdot \right\|}_{2}$ is the $l^{2}-norm$, $B_{x_{R}}$ is the reference block for $x_{R}\in X$, $B_{x}$ is the block for x∈X, and $N_{1}^{ht}$ indicates the length of the image. If $Z_{x_{R}}$ and $Z_{x}$ do not overlap, this distance can be expressed as a chi-squared random variable. The expected values and variance of the distance are expressed as follows:

(2) $$E\left\{Dist^{noisy}\left(B_{x_{R}},\;B_{x}\right)\right\}=Dist^{noisy}\left(B_{x_{R}},B_{x}\right)+2\sigma^{2}$$

(3) $$Var\left\{Dist^{noisy}\left(B_{x_{R}},\;B_{x}\right)\right\}=\frac{8\sigma^{4}}{{\left(N_{1}^{ht}\right)}^{2}}+\frac{8\sigma^{2}Dist^{noisy}\left(B_{x_{R}},B_{x}\right)}{{\left(N_{1}^{ht}\right)}^{2}}$$

However, the probability densities of other $Dist^{noisy}\left(B_{x_{R}},\;B_{x}\right)$ are likely to be severely overlapped by a relatively large sigma or small N. To solve this problem, coarse prefiltering is used to measure the distance between two blocks. Coarse prefiltering involves the application of a normalized 2D linear transformation to the blocks and hard thresholding of the obtained coefficient values. The applied distance expression is as follows:

(4) $$Dist\left(B_{x_{R}},B_{x}\right)=\frac{{\left\|\gamma \left(T_{2D}^{ht}\left(B_{x_{R}}\right)\right)-\gamma \left(T_{2D}^{ht}\left(B_{x}\right)\right)\right\|}_{2}^{2}}{{\left(N_{1}^{ht}\right)}^{2}}$$

where γ is a hard thresholding operator, and $T_{2D}^{ht}$ is a normalized 2D linear transformation. The results, grouped into block matching, are expressed as a set containing blocks similar to $B_{x_{R}}$ as follows:

(5) $$S_{x_{R}}^{ht}=\left\{x\in X:d\left(B_{x_{R}},B_{x}\right)\leq \tau_{match}^{ht}\right\}$$

To construct the $N_{1}^{ht}\times N_{1}^{ht}\times \left|S_{x_{R}}^{ht}\right|$-sized 3D block, group $B_{S_{x_{R}}^{ht}}$ is obtained by stacking matched blocks $B_{x\in S_{x_{R}}^{ht}}$. Co-filtering on group $B_{S_{x_{R}}^{ht}}$ was performed in the 3D transformation area. The formula is expressed as follows:

(6) $${\hat{Y}}_{x_{R}}^{ht}=T_{3D}^{ht}{}^{-1}\left(\gamma \left(T_{3D}^{ht}\left(B_{S_{x_{R}}^{ht}}\right)\right)\right)$$

where $T_{3D}^{ht}$ is a 3D transformation, $T_{3D}^{ht}{}^{-1}$ is an inverse 3D transformation, and ${\hat{Y}}_{x_{R}}^{ht}$ is a co-filtered group estimate.

In aggregation, which is the last step of the first stage, the basic estimate of the actual image is calculated using a weighted analysis of all the overlapped block unit estimates. The weight values for the group estimates are as follows:

(7) $$w_{x_{R}}^{ht}=\left\{\begin{array}{lll} \frac{1}{\sigma^{2}N_{ht}^{x_{R}}}, & & if\;N_{ht}^{x_{R}}\geq 1\\ 1, & & otherwise \end{array}\right.$$

where $N_{ht}^{x_{R}}$ is the number of non-zero coefficients after hard thresholding.

The overall basic estimate, ${\hat{y}}^{basic}$, is available as the weighted average of the block-unit estimate.

(8) $${\hat{y}}^{basic}\left(x\right)=\frac{\sum_{x_{R}\in X}\sum_{x_{m}\in S_{x_{R}}^{ht}}w_{x_{R}}^{ht}{\hat{Y}}_{x_{m}}^{ht,x_{R}}\left(x\right)}{\sum_{x_{R}\in X}\sum_{x_{m}\in S_{x_{R}}^{ht}}w_{x_{R}}^{ht}\kappa_{x_{m}}\left(x\right)}$$

where $\kappa_{x_{m}}:X\to \left\{0,\;1\right\}$ is the characteristic function of the square support in blocks located at $x_{m}\in X$.

In the second stage, improved grouping and co-Wiener filtering were performed using the basic estimates obtained in the first stage. Because the noise in ${\hat{y}}^{basic}$ is assumed to be significantly reduced, the distance is replaced by the square of the normalized $l^{2}-norm$ calculated within the underlying estimate. The block set for co-Wiener filtering is calculated as follows:

(9) $$S_{x_{R}}^{wien}=\left\{x\in X:\frac{{\left\|{\hat{Y}}_{x_{R}}^{basic}-{\hat{Y}}_{x}^{basic}\right\|}_{2}^{2}}{{\left(N_{1}^{wien}\right)}^{2}}\leq \tau_{match}^{wien}\right\}$$

where set $S_{x_{R}}^{wien}$ is used to divide ${\hat{Y}}_{S_{x_{R}}^{wien}}^{basic}$ and $B_{S_{x_{R}}^{wien}}$ into two groups.

The Wiener shrinkage coefficients can be obtained as follows:

(10) $$W_{S_{x_{R}}^{wien}}=\frac{{\left|T_{3D}^{wien}\left({\hat{Y}}_{S_{x_{R}}^{wien}}^{basic}\right)\right|}^{2}}{{\left|T_{3D}^{wien}\left({\hat{Y}}_{S_{x_{R}}^{wien}}^{basic}\right)\right|}^{2}+\sigma^{2}}$$

The co-Wiener filtering of $B_{S_{x_{R}}^{wien}}$ is implemented as an element-wise multiplication of the 3D transform coefficient $T_{3D}^{wien}\left(B_{S_{x_{R}}^{wien}}\right)$ of noisy data with Wiener shrinkage coefficient $W_{S_{x_{R}}^{wien}}$. Subsequently, the inverse transform, *T*, produces the following group estimates:

(11) $${\hat{Y}}_{S_{x_{R}}^{wien}}^{wien}=T_{3D}^{wien}{}^{-1}\left(W_{S_{x_{R}}^{wien}}T_{3D}^{wien}\left(B_{S_{x_{R}}^{wien}}\right)\right)$$

In step 2, aggregation is performed similarly to step 1, resulting in the final estimate, ${\hat{y}}^{final}$. The weight value applied to each $x_{R}\in X$ is expressed as follows:

(12) $$w_{x_{R}}^{wien}=\sigma^{-2}{\left\|W_{S_{x_{R}}^{wien}}\right\|}_{2}^{2}$$

Finally, ${\hat{y}}^{final}$ can be obtained using the weights obtained as follows:

(13) $${\hat{y}}^{final}\left(x\right)=\frac{\sum_{x_{R}\in X}\sum_{x_{m}\in S_{x_{R}}^{wien}}w_{x_{R}}^{wien}{\hat{Y}}_{x_{m}}^{wien,x_{R}}\left(x\right)}{\sum_{x_{R}\in X}\sum_{x_{m}\in S_{x_{R}}^{wien}}w_{x_{R}}^{wien}\kappa_{x_{m}}\left(x\right)}$$

#### 2.2.2. Halo Effect Elimination

**Figure 3:**
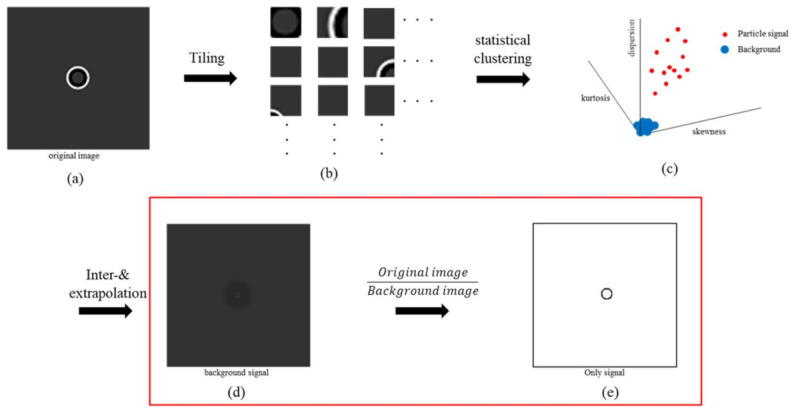
Background correction algorithm. (**a**) Original image, (**b**) sub-image “Tiles,” (**c**) divide two clusters using K-means clustering, (**d**) only background signal, and (**e**) divide original image into background image.

Figure 1b depicts the process for removing the halo effect, which has been the focus of this study. Statistical classification of the signal characteristics of each grid is performed using image patch grids that show only the background or categorize the signal characteristics of each grid as a mixture of background and background pixels. Thereafter, the K-means clustering method [42] was employed to classify a given set of data using a predetermined number of clusters to estimate the background signal and correct the halo signal as a background signal. The detailed process is illustrated in Figure 3.

**Figure 4:**
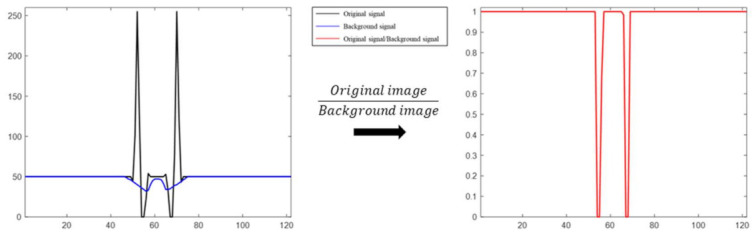
Cell signal after background correction.

The image array, I(x,t), of 255 bits is divided by the sub-image ‘tile’, as shown in Figure 3b. The intensity distribution of the tiles differs between the signal and the background. These tiles are divided using statistical classification and returned to two clusters using the K-means clustering method. As shown in Figure 3c, background tiles are collected in low-density volumes because they are almost identical in statistical distribution. The mean intensity of the tiles that are classified as belonging to the background is defined as the background image, B(x,t), and the original image, I(x,t), is divided by B(x,t). Steps (d) and (e) in Figure 3 are shown in Figure 4. In I(x,t) and B(x,t),the signal corresponding to the cell in I(x,t) is smaller than 1 and closer to 0 because the signal that corresponds to the cell in I(x,t) is smaller than B(x,t). The signal corresponding to the halo of I(x,t) has a value greater than B(x,t); hence, it is divided by a value greater than 1, and a value greater than 1 is specified as a signal similar to the background signal. When this result is imaged, as shown in Figure 3e, the background and image of the cell morphology with the halo effect removed are derived.

#### 2.2.3. Edge Detection of Cells

As shown in Figure 1c, an active contour method was used to detect the shape of a cell in the image that had been removed from the halo effect. Localizing region-based active contour [43] was applied to develop the method devised by Chan–Vese [44]. The total governance formula can be expressed as follows:

(14) $$\begin{array}{cc} E\left(C_{1},C_{2},\phi \right)=\mu & \int \delta \left(\phi \right)\left|\nabla \phi \right|dx+\nu \int H\left(\phi \right)dx\\ & +\lambda_{1}\int {\left|u_{0}-C_{1}\right|}^{2}H\left(\phi \right)dx+\lambda_{2}\int {\left|u_{0}-C_{2}\right|}^{2}\left(1-H\left(\phi \right)\right)dx \end{array}$$

(15) $$C_{1}\left(\phi \right)=\frac{\int u_{0}\left(x\right)H\left(\phi \left(x\right)\right)dx}{\int H\left(\phi \left(x\right)\right)dx},\;C_{2}\left(\phi \right)=\frac{\int u_{0}\left(x\right)\left(1-H\left(\phi \left(x\right)\right)\right)dx}{\int \left(1-H\left(\phi \left(x\right)\right)\right)dx}$$

(16) $$\frac{\partial \phi }{\partial t}=\delta \left(\phi \right)\left\{\mu \nabla \cdot \left(\frac{\nabla \phi }{\left|\nabla \phi \right|}\right)-\nu -\lambda_{1}{\left(u_{0}-C_{1}\right)}^{2}+\lambda_{2}{\left(u_{0}-C_{2}\right)}^{2}\right\}=0$$

The Chan–Vese method is simplified into a stair function in the Mumford–Shah [45] method, and the level set method is applied. $C_{1}$ and $C_{2}$ are the mean values of intensity inside and outside and are defined by formula (15). As shown in Equation (16), the active contour finds that the energy is minimal, which is the boundary of an object.

In the Chan–Vese method, the method developed based on the localized region is expressed as follows:

(17) $$C_{1}\left(\phi \right)=\frac{\int B\left(x,y\right)u_{0}\left(x\right)H\left(\phi \left(x\right)\right)dx}{\int B\left(x,y\right)H\left(\phi \left(x\right)\right)dx},\;C_{2}\left(\phi \right)=\frac{\int B\left(x,y\right)u_{0}\left(x\right)\left(1-H\left(\phi \left(x\right)\right)\right)dx}{\int B\left(x,y\right)\left(1-H\left(\phi \left(x\right)\right)\right)dx}$$

(18) $$B\left(x,y\right)=\left\{\begin{array}{cc} 1, & \left|x-y\right|<r\\ 0, & otherwise \end{array}\right.$$

As the active contour converges, it is calculated by considering only the regions within the r range at the initial mask image boundary as a function of B(x,y). In Figure 1d, the mass center was calculated by considering all of the calculated cell boundary coordinates to the internal coordinates, and this was taken as the center point of the cell. If the center points of the cell images are crossed over time, the path of cell movement can be identified, as shown in Figure 1e. This process is automatically performed in one parameter setting.

## 3. Results and Discussion

After obtaining microscopic images, we performed denoising, halo effect removal, and segmentation for tracking the cells with the MATLAB program according to the algorithmic flow chart described in Figure 1. First, we verified the effectiveness of denoising and halo effect removal. Second, we validated how the denoising and halo effect removal, which were performed earlier, affect segmentation. Finally, the proposed method, manual tracking, and active contour method without the halo effect removal were compared to analyze the cell migration.

**Figure 5:**
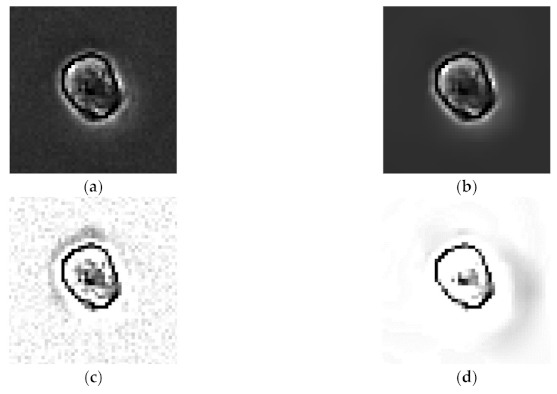
Noise and halo effect removal results: (**a**) Original phase contrast microscopic image. (**b**) Denoised image. (**c**) Halo effect removal without denoising. (**d**) Halo effect removal with denoising.

Figure 5 presents the denoising results obtained when Poisson-Gaussian noise is removed using the block matching 3D method and the halo effect removal results. Figure 5a shows an original phase contrast microscopic image for analyzing cell migration, and Figure 5b presents the denoising results. Whereas the original image presented some noises, the denoised image exhibited noise reduction. However, we note that the halo effect still remained. Therefore, we performed additional halo effect removal. Figure 5c,d present the halo effect removal results with denoising and without denoising to validate the effectiveness of the denoising process.

**Figure 6:**
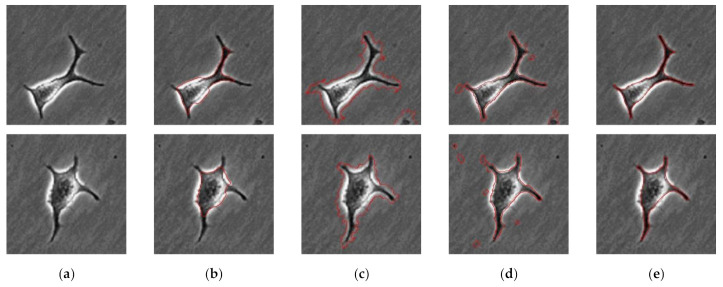
Comparison of segmentation results for different methods. Each column represents the same method results. (**a**) Original phase contrast microscopic image. (**b**) Segmentation result with active contour method. (**c**) EGT method. (**d**) PHANTAST method. (**e**) Proposed method.

After preprocessing, we performed segmentation to track the cells. Figure 6 presents the segmentation results compared to other methods. The active contour, EGT, and PHANTAST methods and the proposed methods were used for comparison. All parameters were adjusted to the manual based on the default value. The Figure 6a column shows the original phase contrast microscopic image that has long pseudopodium with noise and halo effect. The segmentation results using the active contour method are shown in Figure 6b. The active contour method could not properly segment the cells with pseudopodium affected by the halo effect. The Figure 6c,d columns present the results of EGT and PHANTAST method. The EGT method roughly segmented the pseudopodium, but due to the halo effect, the cell contours were not properly segmented. Although the PHANTAST method lowered the halo effect, some errors still remain around the cell contour. Moreover, PHANTAST method contains various parameters so that it is hard to optimize the performance. By contrast, the proposed method significantly reduced the halo effect and segmented the cell contours precisely including pseudopodium (see the Figure 6e column).

**Figure 7:**
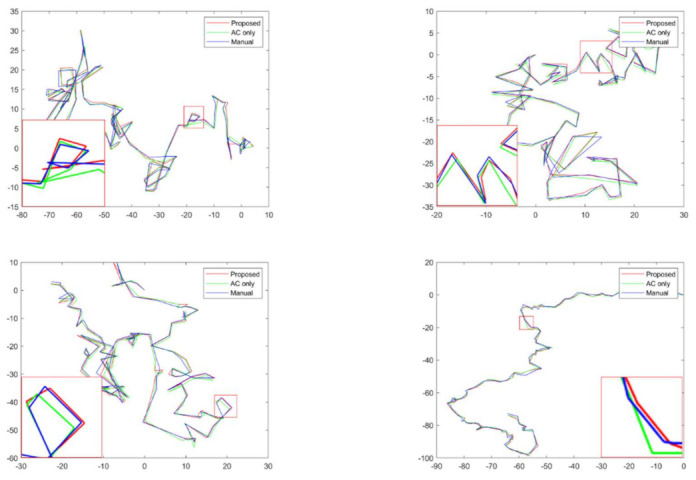
Trajectories of four cells. Each coordinate was represented by normalizing with respect to the starting point. The red line represents the proposed method, the green line represents the method that uses active contour only, and the blue line represents the results tracked manually.

Figure 7 presents the trajectories obtained as the result of tracking the center points of the cells calculated from the detected results. Manual tracking results (blue line) using ImageJ, which were obtained by drawing along the border of the cells with direct hands and calculating the center of mass, were compared with the results of tracking without removing the halo effect (green line) and the methods applied in this study (red line). We observe that the green line has a greater difference from the blue line than the red line.

The results of the comparison of the coordinate values of the trajectories are presented in Table 1. Cell tracking was performed in five cells. A is the difference between the results of manual tracking and the proposed method in the coordinate data at each time step location, and B is the difference between the results of manual tracking and the results of the active contour method only. The average and maximum values of A and B are compared. For the case of the average value of the errors, the result of A exhibited less than 0.5 pixels precision from 0.25 to 0.45 pixels, and B exhibited less precision than A from 0.32 to 0.83 pixels. For the maximum value of the difference, A is from 1.13 pixels to 2.68 pixels and B is from 1.15 pixels to 4.49 pixels. Further, we compared a variance of A and B. The variance of A ranges from 0.04 to 0.17, and the variance of B ranges from 0.06 to 0.39. Using active contour only yields a greater difference from the results of manual tracking, and the results of the proposed method were more accurate.

## 4. Conclusions

In this paper, we present an effective cell segmentation and tracking method for phase-contrast microscopic images. Segmentation and tracking are designed to alleviate noise and halo effects, which are problematic for segmentation using active contours in phase-contrast microscopic images. In the phase-contrast microscopic image, noise was modeled as a Poisson and Gaussian distributions. Therefore, to reduce these noises, noise estimation, Anscombe transform, and BM3D were performed. Additionally, we performed the background correction by solving the hallo effect problem through k-means clustering, a machine learning technique that has strong performance, can be easily implemented and requires less computation. By comparing EGT and PHANTAST, state-of-the-art unsupervised learning methods with the proposed method, we confirmed that our method segments cells effectively considering the pseudopodium. Furthermore, we confirmed that the comparison with manual tracking allows the detection of more accurate boundaries; moreover, we verified the reliability of the algorithm developed in this study. The proposed method outperforms the manual method of directly obtaining cell shapes in terms of time cost of segmentation, and is also highly applicable because it performs with simple algorithms although it is excellent and is performed automatically. It is expected that the cell shape can be accurately detected and utilized for analysis of the motility of the cell considering the shape, rather than analysis via simple location tracking. In addition, the halo effect removal algorithm presented in this study can be employed for cell counting or morphology in phase-contrast microscopic images.

However, in this study, there is a limitation that it is applied only to a single cell, and if there is a circular object, it is difficult to segment it well since the initial point is located by the circular detection algorithm. In a future study, we will apply the algorithm developed in this study to cell images of various types and shapes to optimize them so that they can be used more diversely and generally.

## Figures and Tables

**Table 1:** Comparison of the proposed work and without background correction.

	Parameter	A = |Manual − Proposed Method|			B = |Manual − Active Contour Only|		
Cell Number							
		Average (Pixel)	Max Diff. (Pixel)	Variance (Pixel)	Average (Pixel)	Max Diff. (Pixel)	Variance (Pixel)
1		0.435	2.185	0.164	0.831	2.974	0.331
2		0.332	1.607	0.088	0.560	1.847	0.150
3		0.372	1.893	0.128	0.634	2.090	0.120
4		0.257	1.139	0.043	0.322	1.159	0.067
5		0.428	2.682	0.169	0.771	4.495	0.398
Average		0.364	1.901	0.118	0.623	2.513	0.213

## References

[1] Lauffenburger D.A., Horwitz A.F. (1996). Cell migration: A physically integrated molecular process. Cell.

[2] Franz C.M., Jones G.E., Ridley A.J. (2002). Cell migration in development and disease. Dev. Cell.

[3] Masuzzo P., Van Troys M., Ampe C., Martens L. (2016). Taking aim at moving targets in computational cell migration. Trends Cell Biol..

[4] Theveneau E., Mayor R. (2012). Neural crest delamination and migration: From epithelium-to-mesenchyme transition to collective cell migration. Dev. Biol..

[5] Huda S., Weigelin B., Wolf K., Tretiakov K.V., Polev K., Wilk G., Iwasa M., Emami F.S., Narojczyk J.W., Banaszak M. (2018). Lévy-like movement patterns of metastatic cancer cells revealed in microfabricated systems and implicated in vivo. Nat. Commun..

[6] Debeir O., Adanja I., Kiss R., Decaestecker C. (2008). Models of cancer cell migration and cellular imaging and analysis. Motile Actin Syst. Health Dis..

[7] Li L., Nørrelykke S.F., Cox E.C. (2008). Persistent cell motion in the absence of external signals: A search strategy for eukaryotic cells. PLoS ONE.

[8] Krummel M.F., Bartumeus F., Gérard A. (2016). T cell migration, search strategies and mechanisms. Nat. Rev. Immunol..

[9] Mogilner A., Keren K. (2009). The shape of motile cells. Curr. Biol..

[10] Li H., Qi S., Jin H., Qi Z., Zhang Z., Fu L., Luo Q. (2015). Zigzag generalized levy walk: The in vivo search strategy of immunocytes. Theranostics.

[11] Bise R., Kanade T., Yin Z., Huh S.-i. Automatic cell tracking applied to analysis of cell migration in wound healing assay. Proceedings of the 2011 Annual International Conference of the IEEE Engineering in Medicine and Biology Society.

[12] Acton S.T., Wethmar K., Ley K. (2002). Automatic tracking of rolling leukocytes in vivo. Microvasc. Res..

[13] Jiang R.M., Crookes D., Luo N., Davidson M.W. (2010). Live-cell tracking using SIFT features in DIC microscopic videos. IEEE Trans. Biomed. Eng..

[14] Ebata H., Yamamoto A., Tsuji Y., Sasaki S., Moriyama K., Kuboki T., Kidoaki S. (2018). Persistent random deformation model of cells crawling on a gel surface. Sci. Rep..

[15] Ruprecht V., Wieser S., Callan-Jones A., Smutny M., Morita H., Sako K., Barone V., Ritsch-Marte M., Sixt M., Voituriez R. (2015). Cortical contractility triggers a stochastic switch to fast amoeboid cell motility. Cell.

[16] Roumier A., Olivo-Marin J.C., Arpin M., Michel F., Martin M., Mangeat P., Acuto O., Dautry-Varsat A., Alcover A. (2001). The membrane-microfilament linker ezrin is involved in the formation of the immunological synapse and in T cell activation. Immunity.

[17] Mesquita D., Dias O., Amaral A., Ferreira E. (2010). A comparison between bright field and phase-contrast image analysis techniques in activated sludge morphological characterization. Microsc. Microanal..

[18] Zernike F. (1942). Phase contrast, a new method for the microscopic observation of transparent objects part II. Physica.

[19] Liang C.-C., Park A.Y., Guan J.-L. (2007). In vitro scratch assay: A convenient and inexpensive method for analysis of cell migration in vitro. Nat. Protoc..

[20] Ersoy I., Bunyak F., Mackey M.A., Palaniappan K. Cell segmentation using Hessian-based detection and contour evolution with directional derivatives. Proceedings of the 2008 15th IEEE International Conference on Image Processing.

[21] Zhi X.-H., Meng S., Shen H.-B. (2018). High density cell tracking with accurate centroid detections and active area-based tracklet clustering. Neurocomputing.

[22] Essa E., Xie X. (2018). Phase contrast cell detection using multilevel classification. Int. J. Numer. Methods Biomed. Eng..

[23] Wang W., Taft D.A., Chen Y.-J., Zhang J., Wallace C.T., Xu M., Watkins S.C., Xing J. (2019). Learn to segment single cells with deep distance estimator and deep cell detector. Comput. Biol. Med..

[24] Ambriz-Colin F., Torres-Cisneros M., Avina-Cervantes J., Saavedra-Martinez J., Debeir O., Sanchez-Mondragon J. Detection of Biological Cells in Phase-Contrast Microscopy Images. Proceedings of the 2006 Fifth Mexican International Conference on Artificial Intelligence.

[25] Huh S., Bise R., Chen M., Kanade T. (2010). Automated mitosis detection of stem cell populations in phase-contrast microscopy images. IEEE Trans. Med. Imaging.

[26] Thirusittampalam K., Hossain M.J., Ghita O., Whelan P.F. (2013). A novel framework for cellular tracking and mitosis detection in dense phase contrast microscopy images. IEEE J. Biomed. Health Inform..

[27] Wang Y., Zhang Z., Wang H., Bi S. (2015). Segmentation of the clustered cells with optimized boundary detection in negative phase contrast images. PLoS ONE.

[28] Debeir O., Van Ham P., Kiss R., Decaestecker C. (2005). Tracking of migrating cells under phase-contrast video microscopy with combined mean-shift processes. IEEE Trans. Med. Imaging.

[29] Chalfoun J., Majurski M., Peskin A., Breen C., Bajcsy P., Brady M. (2015). Empirical gradient threshold technique for automated segmentation across image modalities and cell lines. J. Microsc..

[30] Binici R.C., Şahin U., Ayanzadeh A., Töreyin B.U., Önal S., Okvur D.P., Özuysal Ö.Y., Ünay D. Automated segmentation of cells in phase contrast optical microscopy time series images. Proceedings of the 2019 Medical Technologies Congress (TIPTEKNO).

[31] Tsai H.-F., Gajda J., Sloan T.F., Rares A., Shen A.Q. (2019). Usiigaci: Instance-aware cell tracking in stain-free phase contrast microscopy enabled by machine learning. SoftwareX.

[32] Jaccard N., Szita N., Griffin L.D. (2017). Segmentation of phase contrast microscopy images based on multi-scale local basic image features histograms. Comput. Methods Biomech. Biomed. Eng. Imaging Vis..

[33] Jaccard N., Griffin L.D., Keser A., Macown R.J., Super A., Veraitch F.S., Szita N. (2014). Automated method for the rapid and precise estimation of adherent cell culture characteristics from phase contrast microscopy images. Biotechnol. Bioeng..

[34] Ramapraba P., Chitra M., Prem Kumar M. (2017). Effective lesion detection of colposcopic images using active contour method. Biomed. Res..

[35] Chen X., Williams B.M., Vallabhaneni S.R., Czanner G., Williams R., Zheng Y. Learning active contour models for medical image segmentation. Proceedings of the IEEE/CVF Conference on Computer Vision and Pattern Recognition.

[36] Bensch R., Ronneberger O. Cell segmentation and tracking in phase contrast images using graph cut with asymmetric boundary costs. Proceedings of the 2015 IEEE 12th International Symposium on Biomedical Imaging (ISBI).

[37] Yuan C., Yang H. (2019). Research on K-value selection method of K-means clustering algorithm. J. Multidiscip. Res..

[38] Ghane N., Vard A., Talebi A., Nematollahy P. (2017). Segmentation of white blood cells from microscopic images using a novel combination of K-means clustering and modified watershed algorithm. J. Med. Signals Sens..

[39] Foi A., Trimeche M., Katkovnik V., Egiazarian K. (2008). Practical Poissonian-Gaussian noise modeling and fitting for single-image raw-data. IEEE Trans. Image Process..

[40] Anscombe F.J. (1948). The transformation of Poisson, binomial and negative-binomial data. Biometrika.

[41] Dabov K., Foi A., Katkovnik V., Egiazarian K. Image denoising with block-matching and 3D filtering. Proceedings of the Image Processing: Algorithms and Systems, Neural Networks, and Machine Learning.

[42] Xu R., Wunsch D. (2005). Survey of clustering algorithms. IEEE Trans. Neural Netw..

[43] Lankton S., Tannenbaum A. (2008). Localizing region-based active contours. IEEE Trans. Image Process..

[44] Chan T.F., Vese L.A. (2001). Active contours without edges. IEEE Trans. Image Process..

[45] Mumford D.B., Shah J. (1989). Optimal approximations by piecewise smooth functions and associated variational problems. Commun. Pure Appl. Math..

